# Effects of Short-Term Exposure to Inhalable Particulate Matter on Telomere Length, Telomerase Expression, and Telomerase Methylation in Steel Workers

**DOI:** 10.1289/ehp.1002486

**Published:** 2010-12-17

**Authors:** Laura Dioni, Mirjam Hoxha, Francesco Nordio, Matteo Bonzini, Letizia Tarantini, Benedetta Albetti, Alice Savarese, Joel Schwartz, Pier Alberto Bertazzi, Pietro Apostoli, Lifang Hou, Andrea Baccarelli

**Affiliations:** 1 Laboratory of Environmental Epigenetics, Department of Preventive Medicine and Department of Environmental and Occupational Health, University of Milan and IRCCS Ca’ Granda Maggiore Policlinico Hospital Foundation, Milan, Italy; 2 Department of Clinical Medicine, Nephrology and Health Sciences, University of Parma Medical School, Parma, Italy; 3 Department of Clinical and Experimental Sciences, University of Insubria, Varese, Italy; 4 Exposure, Epidemiology and Risk Program, Department of Environmental Health, Harvard School of Public Health, Boston, Massachusetts, USA; 5 Department of Experimental and Applied Medicine, Occupational Medicine and Industrial Hygiene, University of Brescia, Brescia, Italy; 6 Department of Preventive Medicine, Feinberg School of Medicine, Northwestern University, Chicago, Illinois, USA

**Keywords:** epigenetics, particulate matter, telomerase, telomere length

## Abstract

**Background:**

Shortened leukocyte telomere length (LTL) is a marker of cardiovascular risk that has been recently associated with long-term exposure to ambient particulate matter (PM). However, LTL is increased during acute inflammation and allows for rapid proliferation of inflammatory cells. Whether short-term exposure to proinflammatory exposures such as PM increases LTL has never been evaluated.

**Objectives:**

We investigated the effects of acute exposure to metal-rich PM on blood LTL, as well as molecular mechanisms contributing to LTL regulation in a group of steel workers with high PM exposure.

**Methods:**

We measured LTL, as well as mRNA expression and promoter DNA methylation of the telomerase catalytic enzyme gene [human telomerase reverse transcriptase (*hTERT*)] in blood samples obtained from 63 steel workers on the first day of a workweek (baseline) and after 3 days of work (postexposure).

**Results:**

LTL was significantly increased in postexposure (mean ± SD, 1.43 ± 0.51) compared with baseline samples (1.23 ± 0.28, *p*-value < 0.001). Postexposure LTL was positively associated with PM_10_ (β = 0.30, *p*-value = 0.002 for 90th vs. 10th percentile exposure) and PM_1_ (β = 0.29, *p*-value = 0.042) exposure levels in regression models adjusting for multiple covariates. *hTERT* expression was lower in postexposure samples (1.31 ± 0.75) than at baseline (1.68 ± 0.86, *p*-value < 0.001), but the decrease in *hTERT* expression did not show a dose–response relationship with PM. We found no exposure-related differences in the methylation of any of the CpG sites investigated in the *hTERT* promoter.

**Conclusions:**

Short-term exposure to PM caused a rapid increase in blood LTL. The LTL increase did not appear to be mediated by PM-related changes in *hTERT* expression and methylation.

Epidemiological studies have consistently linked both short- and long-term exposure to ambient particulate matter (PM) with increased morbidity and mortality from cardiovascular disease (Brook et al. 2004; Dockery et al. 1993; Hassing et al. 2009). Increased oxidative stress and inflammation are two major mechanisms involved in mediating PM effects (Chuang et al. 2007; Schwartz 2001; Seaton et al. 1995). Shortening of blood leukocyte telomere length (LTL) has been proposed as a marker of cumulative burden of oxidative stress and inflammation (Bekaert et al. 2007; Fitzpatrick et al. 2007). Recent human studies have shown that long-term exposure to air particles from vehicular traffic or related pollutants is associated with reduced LTL (Hoxha et al. 2009; McCracken et al. 2010). In addition, Valdes et al. (2005) demonstrated a dose-dependent inverse relationship between shortened LTLs and pack-years of smoking. The dose-dependent effects of smoking were replicated by Morla et al. (2006) in a cohort of male smokers with and without chronic obstructive pulmonary disease (COPD). However, experimental studies show that during acute inflammation, telomere length in inflammatory cells undergoes a transient increase, which is believed to contribute to the proliferative capacity and clonal expansion necessary to build up an efficient inflammatory response (Weng et al. 1997). These findings are consistent with *in vitro* results showing that LTL can increase at varying rates during cell division as a result of compensatory mechanisms (Hodes et al. 2002). Taken together, these observations suggest that short-term PM exposures might produce an acute increase in LTL, which may participate in sustaining the inflammatory mechanisms associated with PM health effects. Nonetheless, whether short-term PM exposure affects LTL in exposed humans has yet to be evaluated.

Telomeres are composed of repetitive G-rich sequences (TTAGGG) and an abundance of associated proteins that together form a dynamic cap that protects chromosome ends (Blackburn 2001). The progressive telomere shortening associated with cell division is conducive to cell senescence and reduced proliferative capacity (de Lange 1998). Telomerase is a ribonucleoprotein that can synthesize telomere repeats and thus compensate or reverse telomere loss associated with cell division (Greider 1996). The telomerase complex consists of two essential components: a catalytic unit, telomerase reverse transcriptase (hTERT), and the ubiquitously expressed RNA template, *hTR*. A positive correlation has been found between the amount of *hTERT* mRNA and telomerase activity (Cong and Bacchetti 2000; Li et al. 2003; Yi et al. 1999). The level of *hTERT* mRNA expression has been indicated as a primary mechanism of telomerase regulation (Liu et al. 2004). The *hTERT* 5′ flanking region is located in a CpG island (Guilleret et al. 2002), which is highly methylated in normal tissues (Vera et al. 2008), thus suggesting that *hTERT* mRNA expression might be regulated by DNA methylation.

Work conditions may cause exposure to indoor PM levels that are considerably higher than outdoor ambient concentrations. In industrial settings, workers are usually assigned to specific job tasks that tend to be repeated regularly over time. Differences in work routines may thus determine wide and stable gradients of individual exposure to PM, even among workers in the same work facility. However, the regular weekly schedule made of working days and days of rest determines a cycle of exposures and washouts that can be exploited to evaluate potential short-term effects (Tarantini et al. 2009). Steel workers are exposed to high levels of airborne PM and have been shown to be at higher risk for cardiovascular disease (Andjelkovich et al. 1990). In a group of steel workers exposed to high levels of airborne PM, we measured LTL, as well as mRNA expression and promoter DNA methylation of *hTERT*, to identify the effects of short-term exposures on blood LTL.

## Materials and Methods

### Subjects and study design

In a steel production plant in Brescia in northern Italy, we recruited 63 male workers free of cardiopulmonary disease or cancer. All participants had been working in the current job position for at least 1 year and had worked in the plant for 16 ± 10.1 years (mean ± SD; range, 3–35 years). All participants had a rotating weekly schedule based on a 6-day rotation made of 4 days of work followed by 2 days off.

To investigate short-term effects of PM, we obtained blood samples at two different times: The baseline sample was collected in the morning of the first day of a working week (after 2 days off work) before the beginning of any work activity, and the postexposure sample was collected at the same hour on the fourth day of work, after 3 consecutive days of work. A self-administered questionnaire was used to collect detailed information on lifestyle, drug use, medical conditions, body mass index (BMI), education, and residential history. Records from the factory administrative files were used to extract information on occupational history. Individual written informed consent and approval from the local institutional review board were obtained before the study.

### Exposure assessment

Measures of PM with aerodynamic diameters < 10 μm (PM_10_) and < 1 μm (PM_1_) obtained in each of the 11 work areas of the steel production plant were used to estimate individual exposures. PM_10_ and PM_1_ were measured during the 3 days between the baseline and postexposure blood drawing using a GRIMM 1100 light-scattering dust analyzer (Grimm Technologies, Inc., Douglasville, GA, USA). During the 3 working days between the baseline and the postexposure blood collection, each of the study subjects recorded in a personal log the time he spent in each of the work areas. Individual exposure was calculated as the average of area-specific PM levels weighted by the time spent in each area.

### DNA extraction

We used EDTA tubes to collect 7 mL whole blood that was immediately centrifuged on site at 2,500 rpm for 15 min. The buffy coat (400 μL) was transferred in a cryovial and stored at −20°C until DNA extraction. DNA was extracted using the Wizard Genomic DNA purification kit (Promega, Madison, WI, USA) following the manufacturer’s instructions. Purified DNA was resuspended in the kit hydration solution, quantified, and stored at −20°C until use.

### LTL measurement by quantitative polymerase chain reaction (qPCR)

We measured LTL on buffy coat DNA using the real-time quantitative PCR method developed by Cawthon (2002) with minor adaptations (Hoxha et al. 2009). This method measures the relative LTL in genomic DNA by determining the ratio of telomere repeat copy number to single copy gene copy number (T/S ratio) in experimental samples relative to a reference sample. The telomere and human β-globin PCR mix and the thermal cycling profile for both amplicons were previously described (Hoxha et al. 2009). We used pooled control DNA samples from this same study to create a fresh standard curve, ranging from 8 ng/μL to 0.25 ng/μL, in every telomere and human β-globin PCR run. All samples contained *Escherichia coli* DNA heated at 96°C for 10 min and cooled at room temperature. DNA sample (15 ng) was added to each reaction (final volume, 20 μL). All PCRs were performed in triplicate on a DNA Engine Thermal Cycler Chromo4 (Bio-Rad, Hercules, CA, USA).

The intraassay coefficient of variation for the T/S ratio in the present study was 8.1%. The reverse-transcriptase PCR reaction’s mean efficiency for telomere and human β-globin reactions were 99.5% and 97.5%, respectively.

### RNA extraction and *hTERT* mRNA expression

We used the PAXgene Blood RNA Kit to isolate total RNA from whole blood stabilized in PAXgene Blood RNA tubes (both from Qiagen–PreAnalytix, Hombrechtikon, Switzerland). cDNA was synthesized using a High Capacity RNA-to-cDNA kit (Applied Biosystems, Foster City, CA, USA). *hTERT* expression was analyzed by real-time PCR using the Hs 00972650_m1 TaqMan gene expression assay (Applied Biosystems). All measurements were normalized to the expression of *GAPDH* (glyceraldehyde-3-phosphate dehydrogenase gene), which was measured using the Hs 99999905_m1 TaqMan gene expression assay (Applied Biosystems). All PCR runs were performed in triplicate on a 7900HT Fast Real-Time PCR System (Applied Biosystems). We used the thermal cycling profile and the PCR reaction mix components for both cDNA recommended by the manufacturer (Applied Biosystems). Relative gene expression levels were determined on the basis of the ΔΔCT method according to Livak and Schmittgen (2001). Cycle threshold (CT) values obtained for *hTERT* were first normalized for each sample respective to the endogenous housekeeping gene (CT*_hTERT_* − CT*_GAPDH_* = ΔCT_sample_). Subsequently, the ΔCT value obtained was normalized to an external calibrator (ΔCT_sample_ − ΔCT_calibrator_ = ΔΔCT). A pooled study sample was used as a calibrator. Relative expression levels were calculated as 2^−ΔΔCT^ (Livak and Schmittgen 2001).

### *hTERT* promoter methylation

We performed DNA methylation analyses on bisulfite-treated DNA using a highly quantitative analysis based on PCR pyrosequencing (Bollati et al. 2007; Pavanello et al. 2009). DNA (0.5 μg; concentration, 20 ng/μL) was treated using the EZ DNA Methylation-Gold kit (Zymo Research, Orange, CA, USA) according to the manufacturer’s directions. Final elution was performed with 30 μL M-elution buffer. We developed the assay for telomerase methylation by locating the *hTERT* promoter using Genomatix software (Genomatix Software Inc., Ann Arbor, MI, USA) on chromosome 5 (start = 1346370; end = 1346970; length = 601 bases), and amplified the sequence between positions 1346370 and 1346678. In this assay, we measured percent 5-methylcytosine (5mC) at each of three individual CpG dinucleotide positions (position 1, 1346540; position 2, 1346542; position 3, 1346555) within a CpG island located in the *hTERT* promoter. A 50-μL PCR was carried out in 25 μL GoTaq Green Master mix (Promega), 10 pmol forward primer, 10 pmol reverse primer, 50 ng bisulfite-treated genomic DNA, and water. PCR cycling conditions were 95°C for 60 sec, 60°C for 60 sec, and 72°C for 60 sec for 50 cycles. PCR products were purified and sequenced by pyrosequencing as previously described (Bollati et al. 2007) using 0.3 μm sequencing primer. Primers for the assay were forward AGGTTTTGGATGTTAGGGATTTT, reverse-biotinylated CCACAAAACCCTAAAACTTCTCC, and sequencing GGAGTTGTTTGGGAAT.

### Statistical analysis

Student’s paired *t*-test was used to assess differences between baseline and postexposure measurements. We evaluated the association of the levels of PM (PM_10_ and PM_1_) with LTL, *hTERT* mRNA expression, or *hTERT* promoter methylation (separately at each of the three CpG positions) using simple linear regression models, as well as multivariable models adjusting for age, pack-years of smoking, and percent lymphocytes in the differential blood count. We fitted different sets of models using baseline measures, postexposure measures, or the difference between postexposure and baseline measures of the dependent variables (i.e., LTL, *hTERT* mRNA expression, or *hTERT* promoter methylation). In all models, regression coefficients were computed with ordinary least squares estimators. To compare the magnitude of the associations, we report regression coefficients expressing the increase in LTL corresponding to an increase in PM exposure equal to the difference between the 90th and 10th percentiles of exposure. Outliers were excluded from regression analysis by dropping observations with studentized residuals that were > 3 or < −3. We checked regression assumptions by performing diagnostic tests for each model, which included the Shapiro-Wilk test to verify normality of residuals and the White test to verify the homogeneity of variance of the residuals. A two-sided *p*-value < 0.05 was considered statistically significant. All statistical analyses were performed in SAS (version 9.1.3; SAS Institute Inc., Cary, NC, USA).

## Results

### Study subject characteristics

The mean age of the study subjects was 44 years, with a range of 27–55 years. The 25 study subjects (40%) who were current smokers reported smoking 13.0 ± 7.2 (mean ± SD) cigarettes every day. The mean ± SD BMI of the study participants was 26.5 ± 2.7 kg/m^2^. The average levels of exposure estimated for each subject during the 3 days between the baseline and postexposure blood draws were 262 ± 272 μg/m^3^ (range, 74–1,220) for PM_10_ and 8.0 ± 7.7 μg/m^3^ range, 1.7–30.5) for PM_1_. PM_10_ and PM_1_ exposure levels were highly correlated (*r*^2^ = 0.82).

### Blood leukocyte counts

Table 1 shows the mean values, SDs, and range of the variables measured on the baseline and postexposure samples. Differential blood leukocyte counts showed a moderate, nonstatistically significant increase in the proportion of lymphocytes in the postexposure samples (*n* = 62; 32.0 ± 7.6%) compared with the baseline samples (*n* = 62; 30.7 ± 6.7%; *p* = 0.069). We found no differences in total white blood cell number, percent granulocytes, and percent monocytes (Table 1).

### PM exposure and LTL

**Figure 1:**
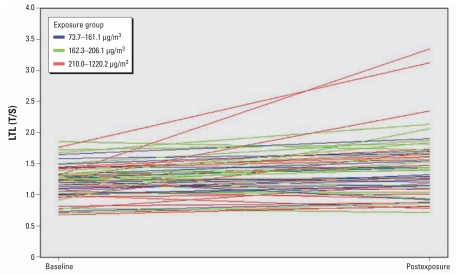
Within-subject changes in LTL between the first day of a workweek (baseline) and after 3 days of work (postexposure). Individuals were categorized according to tertiles of PM_10_ exposure.

LTL showed a significant increase from baseline (*n* = 57; 1.23 ± 0.28 T/S relative units) to postexposure (*n* = 57; 1.43 ± 0.51; *p* < 0.001; Table 1). Figure 1 shows the changes of individuals’ LTL between the first day of a workweek (baseline) and after 3 days of work (postexposure). LTL was significantly increased both in current smokers (*n* = 21; baseline: 1.26 ± 0.30 T/S relative units; postexposure: 1.5 ± 0.58; *p* < 0.0001) and in nonsmokers (*n* = 36; baseline: 1.21 ± 0.27; postexposure: 1.39 ± 0.52; *p* = 0.002). As a sensitivity analysis, we repeated the baseline versus postexposure comparison after excluding the three subjects with the highest postexposure LTL values and found that the increase in LTL was still highly significant (baseline: 1.22 ± 0.28; postexposure: 1.34 ± 0.35; *p* = 0.001).

The levels of individual exposure to PM during the 3 days between the baseline and postexposure blood draws were significantly associated with postexposure LTL in unadjusted regression analysis (for PM_10_, β = 0.26, *p* = 0.002; for PM_1_, β = 0.25, *p* = 0.037), whereas we found no significant associations with baseline LTL (for PM_10_, β = 0.06, *p* = 0.18; for PM_1_, β = 0.02, *p* = 0.71; Table 2). Consequently, levels of individual exposure to PM were associated with the difference in LTL between baseline and postexposure samples (for PM_10_, β = 0.20, *p* = 0.003; for PM_1_, β = 0.22, *p* = 0.016). The results obtained from unadjusted regression models were similar to the results from models adjusted for age, BMI, pack-years of smoking, and percent lymphocytes (Table 2).

### PM exposure and *hTERT* mRNA expression

We measured blood mRNA expression of *hTERT* to gain information on whether the PM-related increase in LTL was produced by telomerase activation. All the blood samples with valid measures had detectable levels of *hTERT* mRNA. *hTERT* mRNA was significantly decreased in postexposure samples (*n* = 58; mean ± SD, 1.31 ± 0.75) compared with baseline samples (*n* = 58; 1.68 ± 0.86; *p* < 0.001; Table 2). However, the levels of individual exposure to PM during the 3 days between the baseline and postexposure blood draws showed no significant associations with *hTERT* mRNA expression in either the baseline or postexposure samples. Also, PM exposure was not associated with the difference in *hTERT* mRNA levels between the baseline and postexposure samples (Table 2).

### PM exposure and *hTERT* promoter methylation

All three of the CpG positions in the *hTERT* promoter that we evaluated showed high levels of methylation, which did not vary between baseline and postexposure samples (Table 1). Mean methylation in position 1 was 93.9% 5mC (range, 90.5–96.8%) at baseline and 93.9% 5mC (range, 89.9–96.8%) in postexposure samples (*p* = 0.84); in position 2, 93.8% 5mC (range, 80.4–100.0%) at baseline and 93.6% 5mC (range, 79.2–97.1%) in postexposure samples (*p* = 0.67); and in position 3, 90.0% 5mC (range, 86.3–95.5%) at baseline and 90.2% 5mC (range, 85.9–93.2%) in postexposure samples (*p* = 0.36; Table 1).

Methylation levels at position 1 were not correlated with *hTERT* mRNA expression at baseline (*r* = −0.12, *p* = 0.37) or in postexposure samples (*r* = −0.15, *p* = 0.28). Methylation levels at position 2 were not correlated with *hTERT* mRNA expression at baseline (−0.06, *p* = 0.65) but showed a significant negative association with *hTERT* mRNA expression in postexposure samples (*r* = −0.48, *p* = 0.0002). Methylation levels at position 3 showed a weak, nonsignificant positive association with *hTERT* mRNA expression at baseline (*r* = 0.24, *p* = 0.07) but not in postexposure samples (*r* = −0.06, *p* = 0.67).

PM exposure showed no consistent associations with *hTERT* methylation at positions 1 and 2, but we observed a negative association of PM with methylation of *hTERT* at position 3 in postexposure samples (unadjusted regression analysis: for PM_10_, β = −0.73, *p* = 0.006; for PM_1_, β = −1.00, *p* = 0.005; multivariable models: for PM_10_, β = 0.70, *p* = 0.017; for PM_1_, β = −1.00, *p* = 0.010, adjusting for age, BMI, education, pack-years, and percent lymphocytes; Table 3).

## Discussion

In the present study of workers in a steel plant with well-characterized measures of exposure to a wide range of PM levels, we found a significant, dose-related increase in LTL after short-term exposure to PM. However, the PM-related increase in LTL did not appear to be explained by modifications of *hTERT* mRNA expression and DNA methylation. The finding of increased LTL in the present study is in contrast with previous reports on long-term PM exposure and LTL. McCracken et al. (2010) reported an inverse association between 1-year exposure levels to black carbon, a tracer of PM from vehicular traffic, and blood LTL in a cohort of elderly individuals in eastern Massachusetts. Hoxha et al. (2009) showed that blood LTL was shorter in police officers with long-term exposure to traffic pollutants than in office workers. The authors speculated that long-term exposure to PM may cause shortened LTL through PM-induced oxidative stress, to which the G-rich structure of telomeres are more sensitive than is normal genomic DNA.

Several experimental *in vitro* models have shown that during acute inflammation, which is a central process in mediating health effects from short-term PM exposure (Brook et al. 2004; Seaton et al. 1995), telomere length increases in inflammatory cells (Weng et al. 1997). These experimental studies have indicated telomerase activation as a potential mechanism determining telomere elongation during acute inflammation (Weng et al. 1995). We therefore further examined *hTERT* mRNA expression and its promoter methylation levels. However, our results do not support a major role for *hTERT* expression in determining increased LTL in human subjects exposed to PM. In fact, we found lower *hTERT* mRNA levels in postexposure samples, which might reflect inactivation by negative feedback of telomerase in the presence of long LTL. However, because the hTERT protein is also posttranscriptionally regulated (Liu et al. 2001), and we did not have a direct measure of telomerase activity in our study, we cannot exclude that telomerase activation had a role in determining increased LTL.

In addition to telomerase, the set of proteins that are associated with telomeres and can affect telomere length has been shown to be extremely complex, and the precise molecular mechanisms that regulate telomere length in lymphocytes or other cell types is not fully understood (Hodes et al. 2002). We did not find consistent associations of PM exposure with *hTERT* mRNA expression and methylation. It is worth noting that previous investigations correlating *hTERT* methylation with its mRNA expression have shown both positive and negative associations (Dessain et al. 2000; Guilleret et al. 2002; Lopatina et al. 2003; Shin et al. 2003). Liu et al. (2004) suggested that those inconsistent correlations may be due to the involvement of a large variety of transcription factors interacting with the *hTERT* promoter. Future studies on PM effects should take into account the complexities of telomerase regulation by evaluating multiple mechanisms of regulation and obtaining direct measures of telomerase activity.

For inflammatory cells, the ability to undergo extensive cell division and clonal expansion is crucial for effectively generating an inflammatory response (Hodes et al. 2002). Cells with shortened telomeres lose their ability to divide and become senescent or undergo apoptosis (Blackburn 2001). In contrast, longer telomeres guarantee the maintenance of cell capacity for rapid proliferation (Hodes et al. 2002). These mechanisms have been shown extensively in lymphocytic subpopulations involved in acute inflammation, including those in peripheral blood (Norrback et al. 1996; Weng et al. 1998). Clonal expansion of circulating leukocytes, which occurs rapidly in subpopulations with longer telomeres, is expected to lead these subpopulations to be more represented in peripheral blood and thus result in an increase in average LTL (Hodes et al. 2002). In our data, we found a moderate, nonsignificant increase in the proportion of circulating blood lymphocytes between baseline and postexposure samples. This increase was not as sharp as that observed for LTL, as expected if only specific lymphocytic subpopulations, such as subpopulations of T or B cells, were to proliferate (Hodes et al. 2002; Weng et al. 1998). Alternatively, migration of less mature leukocytes—which have undergone a lower number of cell divisions and are expected to have longer telomeres—from the bone marrow into the bloodstream might have contributed to increased LTL in postexposure samples. In fact, previous studies have shown that systemic inflammatory responses to PM exposure are associated with the presence of less mature leukocytes in peripheral blood (Suwa et al. 2002; Tan et al. 2000; Terashima et al. 1997). Future studies in PM-exposed subjects should measure LTL after separation of specific leukocyte subpopulations based on their functions and maturity.

Our study design included measurements of LTL, *hTERT* mRNA expression, and *hTERT* methylation in blood leukocyte samples taken on the first and fourth day of a workweek. The two samples were collected to identify short-term fluctuations associated with the weekly cycle of exposure due to working in the plant during the week, followed by exposure cessation during the 2 days off between consecutive weeks. We investigated a population with well-characterized exposure that also allowed for contrasting subjects over a wide range of different exposure levels. Our study was based on subjects working in several work areas of the same factory and did not include a different population of subjects without a specific condition of exposure to inhaled pollutants. However, our primary evaluation of the short-term effects of PM exposure was based on a comparison of paired baseline and postexposure samples from the same subjects, in which each subject served as his own control. Moreover, in the regression models evaluating the dose–response relationships of PM exposure levels with baseline or postexposure measures of LTL, *hTERT* mRNA levels, and *hTERT* methylation, we controlled for potential confounders by fitting multivariable models that included as independent variables several individual characteristics.

Limiting our investigation to individuals working in the same facility avoided potential concerns that are related to the selection of external control subjects, who might have differed from the exposed population in terms of socioeconomic factors and other characteristics determining hiring into the plant (Pearce et al. 2007). Nonetheless, the differences in the personal levels of exposure in our study group were large, providing sufficient contrast for identifying exposure-related associations. For example, the lowest level of PM_10_ observed in our study population (74 μg/m^3^) was only marginally higher than ambient PM_10_ levels measured in the geographic area in which the plant is located [average annual ambient PM_10_ levels between 41 and 57 μg/m^3^ were recorded in the year of the study by different ambient monitoring stations in the Brescia area (Anselmi 2006)], whereas the highest level was 1,220 μg/m^3^. Workers in foundries may have additional exposures in addition to PM, including polycyclic aromatic hydrocarbons (Mirer 1998), carbon monoxide (Lewis et al. 1992), and nonionizing radiation (Gomes et al. 2002). Although the participants in our study were in a modern steel-production facility with state-of-the-art systems for chemical and physical exposure reduction, we cannot exclude that these exposures might have contributed to the observed associations.

We designed our study to take advantage of temporal short-term variations in the exposure due to the weekly cycle of days of work and days off. However, we acknowledge that factors other than the exposure to airborne PM that undergo variations over a week might have influenced the results. For instance, smoking patterns might vary between working and leisure time. We showed that the postexposure increase in LTL was present both in current smokers and in nonsmokers, thus limiting the odds that our findings were driven by recent patterns of active smoking. However, we cannot exclude that other unmeasured risk factors, including recent exposure to passive smoking, influenced our results.

## Conclusions

Our study showed an increase in blood LTL associated with short-term exposure to PM in a group of steel workers. We did not find evidence that the increase in LTL was mediated by changes in *hTERT* mRNA expression and *hTERT* methylation. Future studies are warranted to clarify the mechanisms underlying the increase in LTL induced by short-term PM exposure, as well as its potential roles in mediating PM effects.

## Figures and Tables

**Table 1:** Blood leukocytes counts, LTL, *hTERT* mRNA expression, and *hTERT* promoter methylation measured in 63 foundry workers at baseline and postexposure.

		Baseline		Postexposure		
Outcome	*n*^a^	Mean ± SD	Range	Mean ± SD	Range	*p*-Value
White blood cells (10^3^/mm^3^)	62	7.31 ± 1.6	3.6–10.4	7.3 ± 1.7	4.0–13.0	0.91
Granulocytes (%)	62	58.9 ± 7.2	43.5–76.0	58.2 ± 7.6	44.6–80.6	0.23
Lymphocytes (%)	62	30.7 ± 6.7	15.4–44.1	32.0 ± 7.3	15.7–47.0	0.069
Monocytes (%)	62	10.4 ± 3.5	4.5–19.4	9.8 ± 3.5	3.7–17.5	0.22
LTL (T/S ratio)	57	1.23 ± 0.28	0.68–1.86	1.43 ± 0.51	0.72–3.34	< 0.001
*hTERT* expression (2^−ΔΔCT^)	58	1.68 ± 0.86	0.72–4.86	1.31 ± 0.75	0.52–5.55	< 0.001
*hTERT* methylation (% 5mC)						
Position 1	59	93.9 ± 1.0	90.5–96.8	93.9 ± 1.1	89.9–96.8	0.84
Position 2	59	93.8 ± 2.8	80.4–100.0	93.6 ± 3.1	79.2–97.1	0.67
Position 3	59	90.0 ± 1.9	86.3–95.5	90.2 ± 1.5	85.9–93.2	0.36

a The numbers of observations vary because of missing values due to failed laboratory analyses.

**Table 2:** Association of PM_10_ and PM_1_ exposure with LTL and *hTERT* mRNA expression measured in foundry workers at baseline and postexposure.

Dependent variable	Exposure	Unadjusted		Adjusted^a^	
		β^b^ (95% CI)	*p*-Value	β^b^ (95% CI)	*p*-Value
LTL					
Baseline (*n* = 57^c^)	PM_10_	0.06 (−0.03 to 0.16)	0.18	0.08 (−0.03 to 0.19)	0.13
	PM_1_	0.02 (−0.11 to 0.15)	0.71	0.07 (−0.09 to 0.22)	0.38

Postexposure (*n* = 57^c^)	PM_10_	0.26 (0.10 to 0.42)	0.002	0.30 (0.11 to 0.49)	0.002
	PM_1_	0.25 (0.02 to 0.48)	0.037	0.29 (0.01 to 0.57)	0.042

Difference^d^ (*n* = 57^c^)	PM_10_	0.20 (0.07 to 0.33)	0.003	0.23 (0.08 to 0.38)	0.003
	PM_1_	0.22 (0.04 to 0.40)	0.016	0.21 (0.00 to 0.43)	0.051

*hTERT* expression					
Baseline (*n* = 58^c^)	PM_10_	−0.20 (−0.50 to 0.10)	0.18	−0.05 (−0.36 to 0.26)	0.75
	PM_1_	−0.41 (−0.80 to −0.01)	0.042	−0.15 (−0.57 to 0.27)	0.47

Postexposure (*n* = 58^c^)	PM_10_	−0.07 (−0.33 to 0.19)	0.60	−0.02 (−0.30 to 0.26)	0.89
	PM_1_	−0.15 (−0.49 to 0.20)	0.40	−0.01 (−0.39 to 0.38)	0.97

Difference^d^ (*n* = 58^c^)	PM_10_	0.13 (−0.12 to 0.39)	0.30	0.09 (−0.20 to 0.37)	0.54
	PM_1_	0.26 (−0.08 to 0.60)	0.13	0.20 (−0.16 to 0.57)	0.27

a Regression coefficients, 95% confidence intervals (CIs), and *p*-values estimated from multivariable regression models adjusted for age, BMI, pack-years, and percent lymphocytes.

b β for an increment equal to the difference between the 90th and 10th percentiles of exposure (285.96 μg/m^3^ for PM_10_ and 11.05 μg/m^3^ for PM_1_).

c Values were missing for some subjects because of assay failure; statistical analysis was restricted to subjects with nonmissing values in both the baseline and the postexposure samples.

d Postexposure – baseline.

**Table 3:** Association of PM_10_ and PM_1_ exposure with the levels of methylation at three CpG positions in the promoter of *hTERT* at baseline and postexposure.

*hTERT* methylation	Exposure	Unadjusted		Adjusted^a^	
		β^b^ (95% CI)	*p*-Value	β^b^ (95% CI)	*p*-Value
CpG position 1					
Baseline (*n* = 59^c^)	PM_10_	−0.02 (−0.39 to 0.36)	0.93	0.08 (−0.34 to 0.50)	0.71
	PM_1_	−0.08 (−0.58 to 0.42)	0.76	−0.02 (−0.6 to 0.56)	0.95

Postexposure (*n* = 59^c^)	PM_10_	−0.13 (−0.55 to 0.28)	0.53	−0.15 (−0.62 to 0.32)	0.52
	PM_1_	−0.13 (−0.68 to 0.43)	0.65	−0.13 (−0.76 to 0.50)	0.69

Difference^d^ (*n* = 59^c^)	PM_10_	−0.11 (−0.65 to 0.42)	0.67	−0.24 (−0.84 to 0.36)	0.43
	PM_1_	−0.05 (−0.77 to 0.67)	0.89	−0.03 (−0.83 to 0.77)	0.95

CpG position 2					
Baseline (*n* = 59^c^)	PM_10_	0.15 (−0.89 to 1.20)	0.77	0.05 (−1.18 to 1.27)	0.94
	PM_1_	−0.15 (−1.55 to 1.26)	0.83	−0.32 (−2.00 to 1.36)	0.71

Postexposure (*n* = 59^c^)	PM_10_	−0.26 (−1.41 to 0.89)	0.65	−0.43 (−1.75 to 0.89)	0.52
	PM_1_	−0.35 (−1.89 to 1.19)	0.65	−0.81 (−2.58 to 0.97)	0.36

Difference^d^ (*n* = 59^c^)	PM_10_	−0.41 (−1.83 to 1.00)	0.56	−0.57 (−2.27 to 1.12)	0.50
	PM_1_	−0.20 (−2.11 to 1.70)	0.83	−0.33 (−2.59 to 1.93)	0.77

CpG position 3					
Baseline (*n* = 59^c^)	PM_10_	−0.20 (−0.91 to 0.50)	0.56	−0.21 (−1.00 to 0.59)	0.61
	PM_1_	−0.64 (−1.57 to 0.29)	0.18	−0.68 (−1.76 to 0.40)	0.21

Postexposure (*n* = 59^c^)	PM_10_	−0.73 (−1.24 to −0.22)	0.006	−0.70 (−1.26 to −0.13)	0.017
	PM_1_	−1.00 (−1.69 to −0.32)	0.005	−1.00 (−1.76 to −0.25)	0.010

Difference^d^ (*n* = 59^c^)	PM_10_	−0.53 (−1.19 to 0.13)	0.12	−0.55 (−1.30 to 0.20)	0.15
	PM_1_	−0.37 (−1.27 to 0.54)	0.42	−0.21 (−1.22 to 0.81)	0.69

a Regression coefficients, 95% confidence intervals (CIs), and *p*-values estimated from multivariable regression models adjusted for age, BMI, pack-years, and percent lymphocytes.

b β for an increment equal to the difference between the 90th and 10th percentiles of exposure (285.96 μg/m^3^ for PM_10_ and 11.05 μg/m^3^ for PM_1_).

c Values were missing for some subjects because of assay failure; statistical analysis was restricted to subjects with nonmissing values in both the baseline and the postexposure samples.

d Postexposure – baseline.
